# Key Indicators Affecting Hospital Efficiency: A Systematic Review

**DOI:** 10.3389/fpubh.2022.830102

**Published:** 2022-03-14

**Authors:** Ali Imani, Roghayeh Alibabayee, Mina Golestani, Koustuv Dalal

**Affiliations:** ^1^Tabriz Health Service Management Research Center, Health Economics Department, Tabriz University of Medical Sciences, Tabriz, Iran; ^2^Road Traffic Injury Research Center, Tabriz University of Medical Sciences, Tabriz, Iran; ^3^Faculty of Medicine and Health, Al-Farabi Kazakh National University, Almaty, Kazakhstan; ^4^Department of Public Health Sciences, School of Health Sciences, Mid Sweden University, Sundsvall, Sweden

**Keywords:** hospital inputs, hospital outputs, technical efficiency, hospital process, indicators

## Abstract

**Background:**

Measuring hospital efficiency is a systematic process to optimizing performance and resource allocation. The current review study has investigated the key input, process, and output indicators that are commonly used in measuring the technical efficiency of the hospital to promote the accuracy of the results.

**Methods:**

To conduct this systematic review, the electronic resources and databases MEDLINE (*via* PubMed), Scopus, Ovid, Proquest, Google Scholar, and reference lists of the selected articles were used for searching articles between 2010 and 2019. After in-depth reviews based on the inclusion and exclusion criteria, among 1,537 studies, 144 articles were selected for the final assessment. Critical Appraisal Skills Programme (CASP) Checklist was used for evaluating the quality of the articles. The main findings of studies have been extracted using content analysis.

**Results:**

After the final analysis, the Context/Input indicators that were commonly considered by studies in analyzing hospital technical efficiency include different variables related to Hospital Capacity, Structure, Characteristics, Market concentration, and Costs. The Process/Throughput indicators include different variables related to Hospital Activity or services-oriented process Indicators, Hospital Quality-oriented process indicators, and Hospital Educational processes. Finally, the Output/Outcome indicators include different variables related to Hospital Activity-related output variables and Quality-related output/outcomes variables.

**Conclusion:**

This study has identified that it is necessary to mix and assess a set of input, process, and output indicators of the hospital with both quantitative and qualitative indicators for measuring the technical efficiency of hospitals comprehensively.

## Background

Hospitals are a major part of the health system. Measuring hospital efficiency has been an issue of interest among researchers due to the significant increase in costs of hospital services in recent years (1). Despite providing services to a limited population, hospitals account for a major part of the budget of the healthcare system. These centers consume ~50–80% of total health expenditures (2). This issue highlights the importance of creating additional potential resources and effective usage of available resources through patterns of resource allocation and increasing technical efficiency (3).

Various variables have been mentioned as affecting hospital technical efficiency. These variables can be conceptualized in three categories, namely, the input/context, process, and output indicators. It has been suggested that the degree of non-competitiveness in the hospital market, the hospital profit policy, and regulatory pressures could be regarded as major sources of inefficiency in hospitals (4). Grosskopf et al. (5) have shown that 90% of the teaching hospitals were unable to attain the technical efficiency achieved by non-teaching hospitals. Yong and Harris (6) have shown that hospital size is positively related to technical efficiency. However, the hospital occupancy rate was inversely related to hospital efficiency. Therefore, it could be argued that several key variables influence hospital technical efficiency.

In the absence of prior published systematic reviews, it is important to identify the inputs/context, process, and outputs variables commonly used in measuring hospital technical efficiency. Therefore, the current study objective was to extract key input/context, process, and output indicators affecting the measurement of hospital efficiency. The main research question was: “What are the key indicators that affect hospital efficiency?”

## Methods

### Search Strategy

The research question of the study investigated the key indicators that affect hospital efficiency. We searched for relevant studies in five indexed scientific databases, namely, MEDLINE (*via* PubMed), Scopus, Ovid, Proquest, and Google Scholar to identify relevant English-language studies indexed from 2010 to 2022. We also searched through a list of references from selected articles.

Main keywords, including “Hospital Efficiency, ” “Input indicators, ” “output indicators, ” and “Statistic methods” were used to identify relevant studies. Then, the strategy of the main search in Pubmed and Ovid databases was designed and implemented *via* MeSH as follows: Efficiency, Organizational (Mesh)—Hospital bed capacity (Mesh)—Cost and cost analysis (Mesh)—Economics, hospital (Mesh)—hospitals, and Incomes (Mesh).

Search strategies were designed and implemented in Scopus and Proquest databases without MeSH as shown below:

[Hospital efficiency^*^ AND (Bed OR Capacity OR Physician OR Organizational OR Staff^*^ OR Costs OR Expenditures OR Teaching) AND (Activity^*^ OR Financial outcomes OR Production OR Efficiency)].

We extended our search by looking through the references of the selected studies in the databases. Moreover, we manually searched the gray literature for potentially relevant articles.

### Inclusion and Exclusion Criteria

The criteria for studies to be included in the review were those studies investigating the relationship between input indicators such as hospital capacity, staff number, and staff salary, and output indicators such as hospital income, hospitalization period, and the number of activities such as the number of admissions and consultation. Studies of hospitals that measure efficiency using a statistical method were also included. Articles were excluded if the efficiency assessment concerns specific hospital units.

### Screening Process

The search in citation databases and manual search resulted in a total of 1,537 articles. Among these, 453 articles were omitted due to duplication. Titles and abstracts were then studied based on the inclusion and exclusion criteria, during which 748 cases were omitted upon title investigation and 174 cases were omitted after studying abstracts. From the remaining 162 articles, full-text investigations were qualitatively analyzed using the critical appraisal skills programme (CASP) tool.

**Figure 1:**
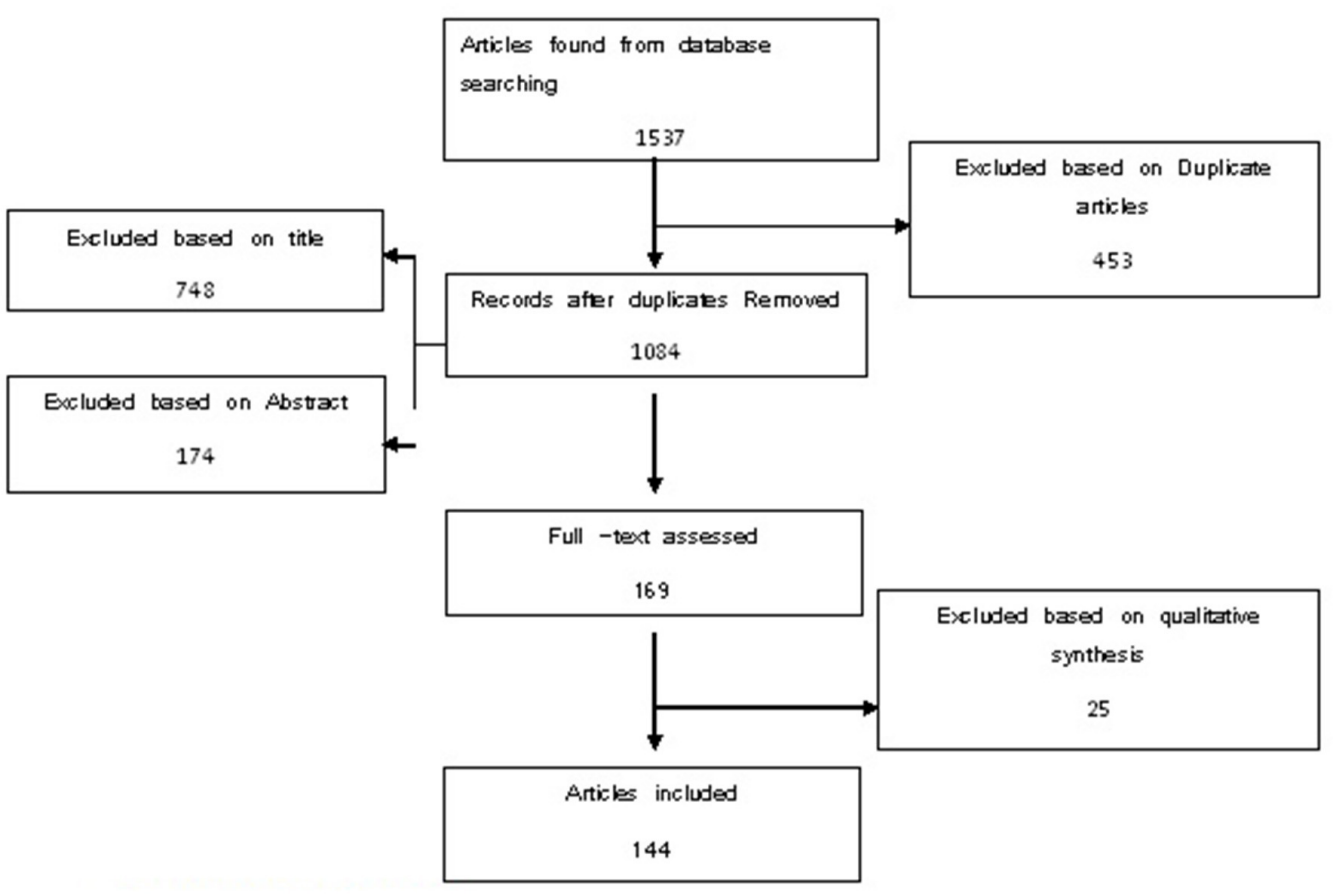
Search and screening process.

A qualitative investigation of articles was conducted in a way that all 169 remaining articles were analyzed independently by two people using the CASP tool for economic studies which included 12 questions. Of 169 remaining article cases, 25 articles had low average credit scores and were excluded from the study. Finally, we begin the content analysis of indicators used in efficiency measurement for the 144 remaining articles. Key details of selected studies were extracted, including title, year of publication, context variables, inputs variables, process variables, outputs variables, and methods used to estimate efficiency. Detailed information on efficiency indicators was also organized using the Context, Input, Process, and Product (CIPP) Model. The details about the search and screening process are shown in Figure 1.

## Results

One hundred and thirty-seven studies fulfilled the inclusion criteria and were all retrospective studies published from 2010 to 2019. Supplementary Table 1 shows a synopsis of the details of all included studies. Of One hundred and thirty-seven investigated articles, key efficiency indicators were divided into three parts, namely, (1) Context/Input indicators, (2) Process/Throughput indicators, and (3) Output/Outcome indicators. The Context/Input indicators included subdivisions of C1. Capacity/labor-related input indicators, C2. Competition-related input indicators, and C3. Hospital expenses related to input indicators. On the other hand, Process/Throughput indicators included P1. Activity/services-oriented process indicators, P2. Quality-oriented process indicators, and P3. Educational process. Finally, Activity-related output indicators and Quality-related output/outcomes indicators are included in Output/Outcome indicators.

The first group of Context/Input indicators is Capacity/labor-related input indicators which are further subdivided into three branches, namely, the number of beds (Actual No. of Open beds/stock of beds/% of skilled beds/No. of ICU), physical space of hospital (Size/Space/equipment/emergency and surgical rooms), and the number of specialties (No. of Employees/No. of FTEs Staff/Skill-Mix Adjustment). The second group of Context/Input indicators is Competition-related input indicators which are subdivided into four branches, namely, Hospital market structure indicators (Herfindahl index (HHI index)/market share/Hirschman-Herfindahl index/firm concentration), Time-related indicators (Average age of patients/physician's Age/On-time start/room turnover times), Hospital production technology indicators (area of specialization/Technological capabilities/high-tech activity Number of high-technology procedures), and Hospital Ownership indicators (teaching hospital Identifier/university hospital identifier/type of control). Lastly, the third group of Context/Input indicators is the hospital expenses-related inputs indicators which include4 main subdivisions, namely, hospital costs indicators (Total cost/Total variable costs/Total operating expenses/Total operating budget/Net operating cost), Index of case-mix (case-mix adjustment/Index/technology index/Service Complexity/Facility service mix), Cost of Labor indicators (Salaries/price of labor/staff hourly wages/Wage Rates), and Price of capital indicators (interest expenses per bed/ratio of interest charges to current assets/interest rate on debt financing; Supplementary Table 1).

Process/Throughput indicators were divided into three main subgroups: activity/services-oriented process indicators, quality-oriented process indicators, and Research and Educational Process indicators. An activity/services-oriented process indicator includes two groups of indicators: 1. Ambulatory, emergency, and outpatient service indicators (No. of ambulatory and emergency care visits/Outpatient Visits/No. of Diagnostics/Case-mix adjusted outpatient/) and 2. Charge efficiency index (Case-mix adjusted discharged patients/Adjusted Discharges/No. of patient discharges). A quality-oriented process indicator includes four groups of indicators: 1. Time efficiency index [Average length of stay (ALoS)/Average hospitalization time (AHT)/Skilled inpatient days/Adjusted Patient Days/Average hospitalization time (TAT)], 2. Occupied day bed index (No. of inpatient admission/rate of hospital admission/No. of post-admission days), 3. The Operability Index (No. of Inpatient and outpatient surgeries/No. of Surgical procedures/operations and newborns), and 4. Bed turnover rate Index [Bed Occupancy Rate (BOR)/Occupied bed-days (OBD)/Bed Turn over (BTO)/Turn over Interval (TOI)/Beds occupied (BAO)/Use of beds/Running sick on a bed (RSB)]. The last indicator of hospital Process/Throughput indicators is associated with Research and Educational Process activities. This included the number of Empowered Staff/patient's training/impact-weighted scientific publications indicators, which were only found in nine studies (3.9%) these indicators had been used as Process/Throughput indicators (Supplementary Table 1).

Output/Outcome indicators have been divided into two main subgroups, namely, Activity-related output indicators and Quality-related output/outcomes indicators. Activity-related output indicators include two groups of indicators: 1. Service mix adjustment (No. of deliveries/No. of services/No. of diagnostic and special services/No. of treated patients/Adjusted Visits/No. of episodes) and 2. Hospital Financial Index [Hospital Revenue/Profit/Outpatient Revenue/The operating margin (OM)/the return on assets (ROA)]. Quality-related output/outcomes indicators include five groups of indicators, namely, (i) Readmission Rates index (Unplanned readmission Rates/Readmission rate for admissions/Case-mix adjusted hospital re-admission), (ii). Total Quality Management (Ratio of births to admissions/Maternal and child health cases), (iii) Utilization Quality Index [Cesarean rate/No. of inappropriate ordinary Discharges and day-hospital admissions/Bed utilization Ratio (BUR)], (iv) Patient Safety Index (Infections due to Medical Care/Postoperative side effects/Accidental Puncture), and (v) Survival and performance Index (Life Expectancy/Mortality Rate/No. of surgeries ratio/Death rate).

## Discussions

To our knowledge, this systematic review is the first to examine key indicators that are commonly used for hospital technical efficiency assessment from a system thinking perspective. As the results of this study show, Context/Input indicators and Process/Throughput indicators were seen to be prevalent in most studies that signify the intent of most hospitals to minimize inputs/processes given a target level of outputs/outcomes in efficiency assessment. Also, as shown in previous research, hospital managers and policymakers have more control over inputs than they have over outputs (7, 8).

Lastly, it was observed that Capacity/labor-related input indicators (57.89%) and Activity/services-oriented process indicators (42.33%) were mostly applied for hospital efficiency measurement. Repeated use of this process/throughput indicator shows its importance in measuring the efficiency and profitability of a hospital. Studies investigating the association between the number of beds and hospital profitability have concluded that hospitals with higher bed numbers have lower efficiency compared with other hospitals. These studies have estimated profitability milestones in the range of 223–503 beds (9–11).

Adhikari et al. (12) identified seven key performance indicators for low-and middle-income countries (LMICs). These included total inpatient days, recurrent expenditure per inpatient day, average length of stay (ALS), infection prevention rate, bed occupancy rate (BOR), inpatient days per technical workforce, and unit cost of outpatient care. One of the other important indicators used in most of the articles (20.01%) is indicator related to Context/Input indicators, where the indicator of the number of beds (86.13%) and the number of specialties (79.56%) have been implemented more than other Context/Input indicators. Results of different studies show that medical staff is considered to be an important input factor in the production function of hospitals as they determine the quality and quantity of hospital output (13). There are several studies investigating resource proportion among personnel groups, and it can affect efficiency and quality in hospitals. This resource proportion mostly includes medical ratios or nursing posts to total staff or beds and medical ratios of staff to nurses (9). Among these studies, Jarman et al. reported that a decrease in medical staff has a direct association with an increase in in-hospital mortality rate. Some other studies also found an association between the level of higher education among nurses and the higher presence of nursing on the one hand and lower mortality and infection rates on the other hand (14, 15). Another indicator that is of importance is the number of hospital specialties. Results of investigated studies show that in addition to educational activities, hospitals with higher specialties are more costly than other hospitals (16). Despite this, the results of another study show that there is no evidence that hospital with higher specialties has lower efficiency compared with other hospitals (17).

Results of the study reveal that Output/Outcome indicators associated with monetary value and quality assessment of hospital performance have been lesser used due to difficulty in collecting statistics and numbers compared to other indicators. It has been shown by Afzali et al. (18), Hollingsworth (19) and Magnussen (20) that many hospital databases suffer from insufficient data regarding a broad range of hospital functions and quality of care, including preventive care, health promotion, and staff development activities.

The current study has two limitations. First, we only used five databases—MEDLINE (*via* PubMed), Scopus, Ovid, Proquest, and Google Scholar—for the published literature. However, these databases are the five most comprehensive in medicine and health economics, and we have complemented the search with complementary hand searches of the reference lists in selected studies. Second, we only included studies that have their full text in English due to the time limitations for translations of non-English publications.

To summarize, we find that to measure the efficiency of a hospital it is necessary to select a combination of quantitative and qualitative indicators to precisely monitor their performance. This can be done by an assessment of a set of input, process, and output indicators of the hospital with qualitative indicators so that a comprehensive and reliable measurement would have resulted. Future research is also required to investigate other indicators behind the hospital allocative efficiency. For example, an investigation of the key input, process, and output allocative efficiency may be effective to discuss the issue.

## Data Availability Statement

The raw data supporting the conclusions of this article will be made available by the authors, without undue reservation.

## Author Contributions

AI contributed to the planning and review supervising. RA and MG contributed to the review. KD contributed to the planning and critical review. All authors contributed to the article writing and have read and approved the manuscript.

## Funding

This study was funded by Tabriz University of Medical Sciences. This study is a part of MSc thesis that has been done with the ethical codeTBZMED.REC.1394.571.

## Conflict of Interest

The authors declare that the research was conducted in the absence of any commercial or financial relationships that could be construed as a potential conflict of interest.

## Publisher's Note

All claims expressed in this article are solely those of the authors and do not necessarily represent those of their affiliated organizations, or those of the publisher, the editors and the reviewers. Any product that may be evaluated in this article, or claim that may be made by its manufacturer, is not guaranteed or endorsed by the publisher.
