# Supplementary material for: Key Indicators Affecting Hospital Efficiency: A Systematic Review
Source: Front Public Health. 2022 Mar 14;10:830102. doi: 10.3389/fpubh.2022.830102 (PMC8964142; doi:10.3389/fpubh.2022.830102)
Supplement: Supplementary file 1 [file Table_1.DOCX]

Table1. Context, Input, Process and Output/Outcome-related (CIPO) indicators

|  |  | **Context/Input variables** | | | | | | | | | | | | **Process/Throughput indicators** | | | | | | | **Output/Outcome indicators** | | | | | | | | |
| --- | --- | --- | --- | --- | --- | --- | --- | --- | --- | --- | --- | --- | --- | --- | --- | --- | --- | --- | --- | --- | --- | --- | --- | --- | --- | --- | --- | --- | --- |
|  |  | **Hospital Capacity/Structure/characteristics/**  **market concentration/Costs** | | | | | | | | | | | | **A1. Activity/services-oriented process**  **indicators** | | **A2. Quality-oriented process**  **indicators** | | | | **A3. Educational Process** | | **Activity- related output indicators** | | **Quality- related output/outcomes**  **indicators** | | | | | |
|  |  | **C1. Capacity/** **labor-related input indicators** | | | **C2. Competition-related input indicators** | | | | | **C3. Hospital expenses-related inputs indicators** | | | |  |  |  |  |  |  |  |  |  |  |  |  |  |  |  |  |
| year | Articles | ***No.of beds*** | ***SIZE*** | ***No.of spec-***  ***Ialty*** | | ***Hospital market*** | ***Time*** | ***Tech*** ***nology*** | ***OWN*** | ***Hospital Coasts*** | ***ICM*** | ***Cost of Labor*** | ***Price of capital*** | **AMO indix** | **Charge index** | **Time index** | **Occupied day bed index** | **The Operability Index**  **(OI)** | **Bed turnover rate (BTR)** | **Research and Educa** **tion** | | **service mix adjustment** | **Hospital Financial Index** | | **Readmission index** | **TQM** | **Utilization**  **Index** | **Patient**  **Safety Index** | **Survival Index** |
|  |  | **(A ctual No. Of**  **Open beds/ stock of beds/ % of skilled beds/ No. of ICU)** | **(Spa ce/ and**  **equipment / emergency/ surgical rooms)** | **(No. Of Employees/ No.of**  **FTEs**  **Staff/ Skill-Mix Adjustment)** | | **share(Herfindahl index(HHI index)/**  **market share/ Hirschman-Herfindahl**  **index/ firm concentration)** | **(Average age of patients/ physician’s**  **Age/**  **On-time start/**  **room**  **turnover times)** | **(area of specialization/ Technological capabilities / high-tech activity\**  **No. of**  **high-technology procedures)** | **(teaching hospital**  **Identifier/university hospital identifier/type of control)** | **(Total cost/**  **Total variable costs/Total operating expenses/ Total operating budget/Net operating cost)** | **(case mix adjustment/**  **Index/**  **technology index/**  **Service Complexity/**  **Facility service mix)** | **(Salaries/ price of labor/ staff hourly wages/ Wage Rates)** | **(interest**  **expenses per bed/ ratio of interest**  **charges to current assets/ interest rate on**  **debt financing)** | **(No.of ambulatory and emergency care visits/ OPt**  **Visits/**  **No. of Diagnostics/ case-mix adjusted outpatient)** | **(Case-mix adjusted discharged patients /Adjusted Discharges/**  **No. of patient discharges)** | **(ALoS/ AHT/Skilled inpatient days \**  **Adjusted Patient Days/**  **TAT)** | **(No.of**  **inpatient admission/**  **rate of hospital admission/**  **No. of post-admission days)** | **(No.of**  **Inpatient and outpatient surgeries/No. Of Surgical procedures/ operations and newborns)** | **(Bed**  **Occupancy**  **Rate/ OBD/ BOR/**  **BTO/**  **TOI**  **BAO/**  **Use of beds/**  **RSB)** | **(No.of Empowered**  **Staff/patienets training/ impact-weighted scientific**  **Publications)** | | **(No. of deliveries/**  **No. of services/**  **No. of diagnostic and special services/**  **No. of treated patients/**  **Adjusted Visits/**  **No. of episodes)** | **(Hospital Revenue**  **Profit/**  **Outpatient Revenue/**  **OM/**  **ROA)** | | **(Unplanned reaadmission Rates/ readmission**  **rate for admissions/**  **case-mix adjusted hospital re-admission)** | **(Ratio of**  **births to admissions/**  **maternal and child health cases)** | **(Caesarean rate)/**  **No. of inappropriate ordinary**  **Discharges andday-hospital**  **Admissions/ BUR)** | **(Infections due to Medical Care/**  **Postoperative side effects/**  **Accidental Puncture)** | **Life**  **Expectancy/**  **Mortality Rate/**  **No. of surgeries ratio/**  **Death rate)** |
| **2010** | **de Castro Lobo, Maria Stella [8]** | * |  | * | |  |  |  |  | * | * |  |  | * |  | * | * |  |  |  | |  |  | |  |  |  |  |  |
|  | **Gai et al [9]** | * |  | * | |  |  |  |  | * |  |  |  | * |  | * |  |  |  |  | |  | * | |  |  |  |  |  |
|  | **Hatam et al[10]** | * |  | * | |  |  |  |  |  |  |  |  |  |  | * | * |  | * |  | |  |  | |  |  |  |  |  |
|  | **Hsieh [11]** | * |  | * | |  |  |  |  | * |  |  |  | * |  |  | * |  |  |  | | * |  | |  |  |  |  |  |
|  | **Jandaghi et al [12]** |  |  | * | |  |  |  |  | * |  |  |  | * |  | * |  |  |  |  | |  |  | |  |  |  |  |  |
|  | **Kristensen T[13]** |  |  |  | |  |  |  |  | * |  |  |  |  |  |  |  |  |  |  | |  | * | |  |  |  |  |  |
|  | **Tlotlego N [14]** | * |  | * | |  |  |  |  |  |  |  |  | * |  | * |  |  |  |  | |  |  | |  |  |  |  |  |
|  | **Dash U[15]** | * |  | * | |  |  |  |  |  |  |  |  | * |  | * |  | * |  |  | | * |  | |  |  |  |  |  |
|  | **Yawe B [16]** | * |  | * | |  |  |  |  |  |  |  |  | * |  | * |  | * |  |  | | * |  | |  |  |  |  |  |
| **2011** | **Al-Shayea AM**  **[17]** |  |  |  | |  |  |  |  |  |  | * |  | * |  | * |  |  | * |  | |  |  | |  |  |  |  |  |
|  | **Bahadori, M [18]** | * |  |  | |  |  |  |  |  |  |  |  |  |  | * |  |  | * |  | |  |  | |  |  |  |  |  |
|  | **Barnum DT [19]** | * |  | * | |  |  |  |  |  |  |  |  | * |  | * |  |  |  |  | |  |  | |  |  |  |  |  |
|  | **Ketabi, S [20]** | * | * | * | |  |  | * |  |  |  |  |  |  |  | * |  |  | * |  | |  |  | |  |  |  |  | * |
|  | **kiadaliri AA[21]** | * |  | * | |  |  |  |  |  |  |  |  | * |  | * |  | * | * |  | |  |  | |  |  |  |  |  |
|  | **Flokou A [22]** | * |  | * | |  |  |  |  |  | * |  |  | * |  | * |  |  |  |  | |  |  | |  |  |  |  |  |
|  | **Herr A[23]** | * |  | * | |  |  |  |  | * |  |  |  |  |  | * |  |  |  |  | |  | * | |  |  |  |  |  |
|  | **Pham TL [24]** | * |  | * | |  |  |  |  |  |  |  |  | * |  |  | * | * |  |  | |  |  | |  |  |  |  |  |
|  | **Shahhoseini R [25]** | * |  | * | |  |  |  |  |  |  |  |  | * |  | * |  | * | * |  | |  |  | |  |  |  |  |  |
|  | **Sahin I [26]** | * |  | * | |  |  |  |  | * |  |  |  | * |  | * |  | * |  |  | |  |  | |  |  |  |  |  |
|  | **Wei CK [27]** | * |  | * | |  |  |  |  |  |  |  |  | * |  | * |  | * |  |  | |  |  | |  |  |  |  |  |
| **2012** | **Kirigia JM [28]** | * |  |  | |  |  |  |  |  |  |  |  |  | * | * |  |  |  |  | |  |  | |  |  |  |  |  |
|  | **Chaabouni S [29]** | * |  | * | |  |  |  |  |  |  |  |  | * |  |  | * |  |  |  | |  |  | | * |  |  |  |  |
|  | **Marnani AB [30]** | * |  | * | |  |  |  |  |  |  |  |  |  |  |  |  | * | * |  | |  |  | |  |  |  |  |  |
|  | **Dimas G [31]** | * |  |  | |  |  |  |  | * |  | * |  | * |  | * |  |  |  |  | |  |  | |  |  |  |  |  |
|  | **Barati B [32]** | * |  | * | |  |  |  |  |  |  |  |  | * |  | * |  |  |  |  | |  |  | |  |  |  |  |  |
|  | **Guerra M [33]** | * |  | * | |  |  |  |  |  |  |  |  |  |  | * | * |  | * |  | |  | * | |  |  |  |  |  |
|  | **Osmani**  **[34]** | * |  | * | |  |  |  |  |  |  |  |  | * |  | * | * |  |  |  | |  |  | |  |  |  |  |  |
|  | **Rahman MA [35]** |  |  |  | |  |  |  |  | * | * |  |  |  |  |  |  |  |  |  | |  | * | |  |  |  |  |  |
|  | **Sheikhzadeh Y [36]** | * |  | * | |  |  |  |  |  |  |  |  | * |  | * |  |  |  |  | |  |  | |  |  |  |  |  |
|  | **Sulku SN [37]** | * |  | * | |  |  |  |  |  |  |  |  | * | * |  |  | * |  |  | |  |  | |  |  |  |  | * |
|  | **Tiemann O [38]** |  |  | * | |  |  |  |  | * |  |  |  |  |  | * |  |  |  |  | |  |  | |  |  |  |  | * |
| **2013** | **Abou El-Seoud M. S. [39]** | * |  | * | |  |  |  |  |  |  |  |  | * |  | * |  |  |  |  | | * |  | |  |  |  |  |  |
|  | **Ajlouni MM[40]** | * |  | * | |  |  |  |  |  |  |  |  |  |  | * |  | * |  |  | |  |  | |  |  |  |  |  |
|  | **Arfa C [41]** | * |  | * | |  |  |  |  | * |  |  |  | * |  |  | * |  |  |  | |  |  | |  |  |  |  |  |
|  | **Audibert M [42]** | * | * | * | |  |  |  |  |  |  |  |  | * |  | * |  |  |  |  | |  |  | |  |  |  |  |  |
|  | **Hatam N [43]** | * |  |  | |  |  |  |  |  |  |  |  |  |  | * | * |  |  |  | |  |  | |  |  |  |  |  |
|  | **Besstremyannaya G [44]** | * |  | * | |  |  |  |  |  |  |  |  | * | * |  |  |  |  |  | |  |  | |  |  |  |  |  |
|  | **Chu HL [45]** |  |  |  | |  |  | * |  | * |  |  |  | * |  |  |  | * |  |  | |  | * | |  |  |  |  |  |
|  | **Ferrier GD [46]** | * |  | * | |  |  |  |  |  |  |  |  | * |  |  | * | * |  |  | |  |  | |  |  |  |  |  |
|  | **Gok MS [47]** | * |  | * | |  |  |  |  |  |  |  |  | * | * | * | * | * |  |  | |  |  | |  |  |  | * |  |
|  | **Gholipour K [48]** | * |  |  | |  |  |  |  |  |  |  |  |  |  | * | * |  |  |  | |  |  | |  |  |  |  |  |
|  | **Tahmasebi N [49]** |  |  | * | |  |  |  |  | * |  |  |  | * | * |  |  |  |  |  | |  |  | |  |  |  |  |  |
|  | **Mitropoulos P [50]** |  |  | * | |  |  |  |  |  |  |  |  | * |  |  | * | * |  |  | | * |  | |  |  |  |  |  |
|  | **Nayar P [51]** | * |  | * | |  |  |  |  | * |  |  |  | * |  | * |  |  |  | * | |  |  | |  |  |  |  |  |
|  | **Yusefzadeh H [52]** | * |  | * | |  |  |  |  |  |  |  |  | * |  |  | * |  |  |  | |  |  | |  |  |  |  |  |
| **2014** | **Adham D [53]** | * |  |  | |  |  |  |  |  |  |  |  |  |  | * | * |  | * |  | |  |  | |  |  |  |  |  |
|  | **Araújo C [54]** | * | * | * | |  |  |  |  |  |  |  |  | * |  | * |  | * |  |  | |  |  | |  |  |  |  |  |
|  | **Goudarzi R [55]** | * |  | * | |  |  |  |  |  |  |  |  |  |  |  | * | * | * |  | |  |  | |  |  |  |  |  |
|  | **Bilsel M [56]** | * |  | * | |  |  |  |  | * |  |  |  | * |  |  | * | * |  |  | |  |  | |  |  |  |  | * |
|  | **Goudarzi R [57]** | * |  | * | |  |  |  |  |  |  |  |  | * |  |  | * |  |  |  | |  |  | |  |  |  |  |  |
|  | **Imamgholi S[58]** | * |  |  | |  |  |  |  |  |  |  |  |  |  | * | * |  | * |  | |  |  | |  |  |  |  |  |
|  | **Jehu-Appiah C [59]** | * |  | * | |  |  |  |  | * |  |  |  | * |  | * |  |  |  |  | | * |  | |  |  |  |  |  |
|  | **Kawaguchi H [60]** | * |  | * | |  |  |  |  | * |  |  | * | * |  | * |  |  |  |  | | * |  | |  |  |  |  |  |
|  | **Kalhor R [61]** | * |  |  | |  |  |  |  |  |  |  |  |  |  | * | * |  | * |  | |  |  | |  |  |  |  |  |
|  | **Li H [62]** | * |  | * | |  |  |  |  |  |  |  |  | * | * |  |  |  |  |  | |  |  | |  |  |  |  |  |
|  | **Lotfi F [63]** | * |  | * | |  |  |  |  |  |  |  |  | * |  | * |  | * |  |  | |  |  | |  |  |  |  |  |
|  | **Mehrtak M [64]** | * |  | * | |  |  |  |  |  |  |  |  |  | * |  |  | * | * |  | |  |  | |  |  |  |  |  |
|  | **Pourmohammadi K [65]** | * |  | * | |  |  |  |  |  |  |  |  | * |  | * |  |  |  |  | |  |  | |  |  |  |  |  |
|  | **Rasool SA [66]** | * |  | * | |  |  |  |  |  |  |  |  | * |  | * |  | * |  |  | |  |  | |  |  |  |  |  |
|  | **Applanaidu SD [67]** | * |  | * | |  |  |  |  |  |  |  |  | * |  | * |  | * |  |  | | * |  | |  |  |  |  |  |
|  | **Torabipour A [68]** | * |  | * | |  |  |  |  |  |  |  |  | * |  | * | * | * |  |  | |  |  | |  |  |  |  |  |
|  | **Yang J [69]** | * |  | * | |  |  |  |  |  |  |  |  | * |  | * |  |  |  |  | |  |  | |  |  |  |  |  |
|  | **Younsi M [70]** | * |  |  | |  |  |  |  |  |  |  |  |  |  | * | * |  | * |  | |  |  | |  |  |  |  |  |
| **2015** | **Atake EH [71]** | * |  | * | |  |  |  |  |  |  |  |  |  |  | * | * | * |  |  | |  |  | |  |  |  |  |  |
|  | **Atilgan E [72]** |  |  |  | |  |  |  |  | * |  |  |  | * |  | * |  |  |  |  | |  |  | |  |  |  |  |  |
|  | **Bwana KM [73]** | * |  | * | |  |  |  |  | * |  |  |  | * |  | * |  | * |  |  | |  |  | |  |  |  |  |  |
|  | **Cheng Z [74]** | * |  | * | |  |  |  |  |  |  |  |  | * |  | * |  |  |  |  | |  |  | |  |  |  |  |  |
|  | **Ekiyor A [75]** | * |  |  | |  |  |  |  |  |  |  |  |  |  | * | * |  |  |  | |  |  | |  |  |  |  |  |
|  | **Ferreira D [76]** | * |  | * | |  |  |  |  | * |  |  |  | * |  | * |  |  |  |  | |  |  | |  |  |  |  |  |
|  | **Gok MS [77]** | * |  | * | |  |  |  |  |  |  |  |  | * | * | * | * | * |  |  | |  |  | |  |  |  | * |  |
|  | **Kittelsen SA[78]** |  |  |  | |  |  |  |  | * |  |  |  | * |  | * |  | * |  |  | |  |  | |  |  |  |  |  |
|  | **Li H [79]** | * |  | * | |  |  |  |  |  |  |  |  | * | * |  |  |  |  |  | |  |  | |  |  |  |  |  |
|  | **Lindlbauer I [80]** | * |  | * | |  |  |  |  | * |  |  |  |  |  |  |  |  |  |  | | * |  | |  |  |  |  |  |
|  | **Golestani M[81]** |  |  |  | |  |  |  |  | * |  |  |  |  |  |  |  | * |  |  | |  |  | |  |  |  |  |  |
|  | **Masoompour SM [82]** | * |  |  | |  |  |  |  |  |  |  |  |  | * |  | * |  |  |  | |  |  | |  |  |  |  |  |
|  | **Matranga D[83]** | * |  | * | |  |  |  |  |  |  |  |  |  | * |  | * |  |  |  | |  |  | |  |  | * |  |  |
|  | **Narcı HÖ [84]** | * |  | * | |  |  |  |  |  |  |  |  | * | * | * |  | * |  |  | |  |  | |  |  |  |  |  |
|  | **Prakash V [85]** | * |  | * | |  |  |  |  |  |  |  |  | * |  |  |  | * |  |  | | * |  | |  |  |  |  |  |
|  | **Takundwa R [86]** | * |  |  | |  |  |  |  |  |  |  |  |  | * | * |  |  |  |  | |  |  | |  |  |  |  |  |
|  | **Rezaee MJ [87]** | * | * | * | |  |  |  |  |  |  |  |  | * |  | * |  |  | * |  | |  |  | |  |  |  |  |  |
|  | **Shetabi HR [88]** | * |  | * | |  |  |  |  |  |  |  |  | * |  | * |  |  | * |  | |  |  | |  |  |  |  |  |
|  | **Xu GC [89]** | * |  | * | |  |  |  |  | * |  |  |  | * | * |  |  |  | * |  | |  |  | |  |  |  |  |  |
| **2016** | **Amole BB [90]** | * |  | * | |  |  |  |  |  |  |  |  | * |  | * |  | * |  |  | |  |  | |  | * |  |  |  |
|  | **Arfa C [91]** | * |  | * | |  |  |  |  | * |  |  |  | * |  |  | * |  |  |  | |  |  | |  |  |  |  |  |
|  | **Atilgan E [92]** | * |  | * | |  |  |  |  |  |  |  |  | * |  |  |  |  |  |  | |  |  | |  |  |  |  |  |
|  | **Atilgan E [93]** | * |  | * | |  |  |  |  |  |  |  |  |  | * | * |  |  |  |  | |  |  | |  |  |  |  |  |
|  | **Büchner VA [94]** |  |  | * | |  |  |  |  | * |  |  |  |  |  | * |  |  |  |  | |  |  | |  |  |  |  |  |
|  | **Chaabouni S [95]** | * |  | * | |  |  |  |  | * |  |  |  | * |  |  | * |  |  |  | |  |  | | * |  |  |  |  |
|  | **Cheng Z [96]** | * |  | * | |  |  |  |  |  |  |  |  | * |  | * |  |  |  |  | | * |  | |  |  |  |  |  |
|  | **Flokou A [97]** | * |  | * | |  |  |  |  |  |  |  |  | * |  | * |  | * |  |  | |  |  | |  |  |  |  |  |
|  | **Fragkiadakis G [98]** |  |  | * | |  |  |  |  | * |  |  |  | * |  | * |  | * |  |  | |  |  | |  |  |  |  |  |
|  | **Hamidi S [99]** | * |  | * | |  |  |  |  |  |  |  |  | * |  | * |  |  |  |  | |  |  | |  |  |  |  |  |
|  | **Hassanain M [100]** |  |  |  | |  | * |  |  |  |  |  |  |  |  |  |  |  |  |  | | * |  | |  |  | * |  |  |
|  | **Isai V [101]** |  |  |  | |  |  |  |  | * |  |  |  | * |  | * |  |  |  |  | |  | * | |  |  |  |  |  |
|  | **Kalhor R [102]** | * |  | * | |  |  |  |  |  |  |  |  | * |  | * | * | * |  |  | |  |  | |  |  |  |  |  |
|  | **Kakeman E[103]** | * |  | * | |  |  |  |  |  |  |  |  | * |  | * |  | * |  |  | |  |  | |  |  |  |  |  |
|  | **Mahate A [104]** | * |  | * | |  |  |  |  |  |  |  |  | * |  | * |  |  |  |  | |  |  | |  |  |  |  |  |
|  | **Mohammadi H [105]** | * |  | * | |  |  |  |  |  |  |  |  |  |  |  |  |  |  |  | |  |  | |  |  |  |  |  |
|  | **Mujasi PN[106]** | * | * | * | |  |  |  | * |  |  |  |  | * |  | * |  |  |  |  | |  |  | |  |  |  |  |  |
|  | **Nabilou B[107]** | * |  | * | |  |  |  |  |  |  |  |  | * |  | * |  | * |  |  | |  |  | |  |  |  |  |  |
| **2017** | **Helal SM[108]** | * |  | * | |  |  |  |  |  |  |  |  | * |  | * |  |  |  |  | | * |  | |  |  |  |  |  |
|  | **Arfa C [109]** | * |  | * | |  |  |  |  | * |  |  |  | * |  |  | * |  |  |  | |  |  | |  |  |  |  |  |
|  | **Choi JH [110]** | * |  | * | |  |  |  |  |  |  |  |  | * |  |  | * |  |  |  | |  |  | |  |  |  |  |  |
|  | **Erus B [111]** | * |  | * | |  |  |  |  |  |  |  |  | * |  | * |  | * |  |  | |  |  | |  |  |  |  |  |
|  | **Farzianpour F [112]** | * |  | * | |  |  |  |  |  |  |  |  | * |  |  |  |  | * |  | |  |  | |  |  |  |  |  |
|  | **Flokou A [113]** | * |  | * | |  |  |  |  |  |  |  |  | * |  | * |  | * |  |  | |  |  | |  |  |  |  |  |
|  | **Jia T [114]** | * |  | * | |  |  |  |  |  |  |  |  | * | * | * |  |  |  |  | |  |  | |  |  |  |  |  |
|  | **Bahreini R [115]** | * | * |  | |  | * |  | * | * |  |  |  |  |  |  |  |  |  |  | |  | * | |  |  |  |  |  |
|  | **Helal SM[116]** | * |  | * | |  |  |  |  |  |  |  |  | * |  | * |  |  |  |  | | * |  | |  |  |  |  |  |
|  | **Ali AM [117]** | * |  | * | |  |  |  |  |  |  |  |  | * |  |  |  |  |  |  | | * |  | |  |  |  |  |  |
|  | **Khushalani J [118]** | * |  | * | |  |  |  |  | * | * |  |  | * |  |  |  | * |  |  | |  |  | |  |  |  |  |  |
|  | **Li NN [119]** | * |  | * | |  |  |  |  | * |  |  |  | * | * | * |  |  |  |  | |  |  | |  |  |  |  |  |
|  | **Mitropoulos P [120]** | * |  | * | |  |  |  |  | * |  |  |  | * | * |  |  |  |  |  | |  |  | |  |  |  |  |  |
|  | **Moradi G [121]** | * |  |  | |  |  |  |  |  |  |  |  |  |  | * | * |  |  |  | |  |  | |  |  |  |  |  |
|  | **Mousa W [122]** | * |  | * | |  |  |  |  |  |  |  |  | * |  | * |  | * |  |  | | * |  | |  |  |  |  |  |
|  | **Raei B [123]** | * |  | * | |  |  |  |  |  |  |  |  |  |  |  | * |  |  |  | |  |  | |  |  |  |  | * |
|  | **Silwal PR [124]** | * |  | * | |  |  |  |  | * |  | * |  | * |  | * |  |  |  |  | |  |  | |  |  |  |  |  |
|  | **Sultan WI[125]** | * |  | * | |  |  |  |  |  |  |  |  | * |  | * |  |  |  |  | |  |  | |  |  |  |  |  |
|  | **Wang ML[126]** | * |  | * | |  |  |  |  |  |  |  |  | * |  | * |  |  |  |  | |  |  | |  |  |  |  |  |
|  | **Xenos P [127]** | * |  | * | |  |  |  |  | * |  |  |  |  | * |  |  |  |  |  | | * |  | |  |  |  |  |  |
| **2018** | **Ferreira DC[128]** | * |  | * | |  |  |  |  | * |  |  |  | * |  | * |  | * |  |  | |  |  | |  |  |  |  |  |
|  | **Lai HP [129]** | * | * | * | | * | * |  |  |  |  |  |  |  |  |  |  |  |  |  | | * |  | |  |  |  |  |  |
|  | **Leleu H [130]** | * |  | * | |  |  |  |  | * | * |  |  | * |  | * | * | * |  |  | |  |  | | * |  |  |  | * |
|  | **Pirani N** **[131]** | * |  | * | |  |  |  |  |  |  |  |  |  |  | * |  |  | * |  | |  |  | |  |  |  |  |  |
|  | **Rezaee MJ[132]** | * |  |  | |  |  |  |  |  |  |  |  |  |  | * | * |  | * |  | |  |  | |  |  |  |  |  |
|  | **Rotea CS [133]** | * |  |  | |  |  |  |  |  | * |  |  |  |  | * |  | * | * |  | |  |  | |  |  |  |  |  |
|  | **Sajadi HS [134]** |  |  |  | |  |  |  |  |  |  |  |  | * |  | * | * | * | * |  | | * |  | |  |  |  |  |  |
|  | **Wei Y [135]** |  |  |  | |  |  |  |  | * |  |  |  | * | * |  |  |  |  |  | |  |  | |  |  | * |  |  |
|  | **Migdadi YK [136]** | * |  | * | |  |  |  |  |  |  |  |  |  |  | * |  | * | * |  | |  |  | |  |  |  |  |  |
|  | **Yildiz MS[137]** | * | * | * | |  |  |  |  |  |  |  | * | * |  | * |  | * | * |  | | * | * | |  |  |  |  |  |
| **2019** | **Alatawi A [138]** | * | * | * | |  |  |  |  |  |  |  |  | * |  | * | * |  | * |  | | * |  | |  |  |  |  |  |
|  | **Hatefi SM[139]** |  |  | * | |  |  |  |  | * |  |  |  |  |  |  |  |  |  |  | |  |  | |  |  |  | * |  |
|  | **Kakemam E [140]** | * |  | * | |  |  |  |  |  |  |  |  | * |  | * |  |  |  |  | |  |  | |  |  |  |  |  |
|  | **Küçük A [141]** | * | * | * | |  |  |  |  | * |  |  |  | * |  | * |  | * |  |  | |  | * | |  |  |  |  |  |
|  | **Huang L [142]** | * | * | * | |  |  |  |  | * |  |  |  | * | * |  |  |  | * |  | |  | * | |  |  |  |  |  |
|  | **Giménez V[143]** |  |  |  | |  |  |  |  | * |  |  |  |  |  |  |  |  |  |  | |  |  | | * |  |  |  |  |
|  | **Yitbarek K [144]** | * |  | * | |  |  |  |  | * |  | * |  |  |  |  |  |  |  |  | | * |  | |  |  |  |  |  |
| **2020** | **Sarabi Asiabar A [158]** | * |  | * | |  |  |  | * |  |  |  | * | * | * |  | * | * |  |  | |  |  | |  | * |  |  |  |
|  | **Nundoochan A [161]** | * |  | * | |  |  |  |  |  |  |  |  | * |  |  | * |  |  |  | |  |  | |  |  |  |  |  |
|  | **Ortega-Díaz MI [162]** | * |  | * | |  |  |  |  | * |  |  |  | * | * |  |  |  |  |  | |  |  | |  |  |  |  |  |
|  | **Alatawi AD[163]** | * |  | * | |  |  |  |  |  |  |  |  | * |  | * |  |  |  |  | |  |  | |  |  |  |  |  |
|  | **Küçük A [164]** | * |  | * | |  |  | * |  |  |  |  | * | * |  | * |  | * |  |  | |  | * | |  |  |  |  |  |
| **2021** | **Han A [159]** | * | * | * | | * |  |  | * | * |  |  |  | * |  | * |  | * |  |  | |  |  | |  |  |  |  |  |
|  | **Goudarzi R [160]** | * |  | * | |  |  |  |  |  |  |  |  | * |  | * |  |  |  |  | |  |  | |  |  |  |  |  |
| ***Indicators Frequency***  ***(n):*** | | **125** | **12** | **116** | | **2** | **3** | **3** | **4** | **46** | **6** | **4** | **4** | **102** | **23** | **95** | **40** | **49** | **26** | **1** | | **22** | **12** | | **4** | **2** | **3** | **3** | **6** |
| ***Indicators Percentage***  ***(%):***  /144 | | **86.8** | **8.3** | **80.5** | | **1.3** | **2.0** | **2.0** | **2.7** | **31.9** | **4.1** | **2.7** | **2.7** | **70.8** | **15.97** | **65.97** | **27.77** | **34.02** | **18.05** | **0.69** | | **15.27** | **8.3** | | **2.7** | **1.3** | **2.0** | **2.0** | **4.1** |

Notes: **Emrg** = Emergency; **OPt** = Out Patient; **INPT**= Inpatient; **Res**=Research;**DEA**= data envelopment analysis; **SFA**= stochastic frontier analysis; **RN**= Registered nurse; **LPN**= licensed practical nurse;**HI**= Herfindahl index; **MI**= Malmquist Indices; **HHI**=Hirschman-Herfindahl Index; **SIZE**= hospital size; **BOR**= Bed Occupancy Rate (Inpatient days / (number of beds a 365)); **TEACH**= Teaching Status; **OWN**= Hospital Ownership; **MRA**=Multi Regression Analysis; **BTO**= Bed Turn Over; **TOI**= Turn over Interval; **OM**= The operating margin; **ROA**= The return on assets; **TAT**= Total asset turnover; **TQM**= Total quality management; **BAO**= Beds actually occupied (days of hospitalization / no. days of the month); **AHT**= Average hospitalization time; **RSB**= Running sick on a bed (total admissions / physical beds); **OI**= The Operability Index (cases operated / cases classified as surgical); **ICM**= Index of case mix (Total weighted cases / Total cases solved x 100); **U%**= Use% (Use beds / no. days of month x 100); **Price of capital**= (Depreciation +interest expenses)/ No.f hospital beds; **Price of labor**= Total annual salaries /FTE employees; **AMO** = Ambulatory, emergency and outpatient service; **BTR**= bed turnover rate; **OBD**= Occupied bed-days; **BUR**= Bed utilization Ratio.
